# Point convolutional neural network algorithm for Ising model ground state research based on spring vibration

**DOI:** 10.1038/s41598-023-49559-3

**Published:** 2024-02-01

**Authors:** Zhelong Jiang, Gang Chen, Ruixiu Qiao, Pengcheng Feng, Yihao Chen, Junjia Su, Zhiyuan Zhao, Min Jin, Xu Chen, Zhigang Li, Huaxiang Lu

**Affiliations:** 1 Institute of Semiconductors, Chinese Academy of Sciences, Beijing, China; 2 Materials and Optoelectronics Research Center, University of Chinese Academy of Sciences, Beijing, China; 3 School of Microelectronics, University of Science and Technology of China, Hefei, China; 4 College of Microelectronics, University of Chinese Academy of Sciences, Beijing, China; 5 Semiconductor Neural Network Intelligent Perception and Computing Technology Beijing Key Laboratory, Beijing, China

**Keywords:** Mathematics and computing, Physics

## Abstract

The ground state search of the Ising model can be used to solve many combinatorial optimization problems. Under the current computer architecture, an Ising ground state search algorithm suitable for hardware computing is necessary for solving practical problems. Inspired by the potential energy conversion of the springs, we propose the Spring-Ising Algorithm, a point convolutional neural network algorithm for ground state search based on the spring vibration model. Spring-Ising Algorithm regards the spin as a moving mass point connected to a spring and establishes the equation of motion for all spins. Spring-Ising Algorithm can be mapped on AI chips through the basic structure of the neural network for fast and efficient parallel computing. The algorithm has shown promising results in solving the Ising model and has been tested in the recognized test benchmark K2000. The optimal results of this algorithm after 10,000 steps of iteration are 2.9% of all results. The algorithm introduces the concept of dynamic equilibrium to achieve a more detailed local search by dynamically adjusting the weight of the Ising model in the spring oscillation model. Spring-Ising Algorithm offers the possibility to calculate the Ising model on a chip which focuses on accelerating neural network calculations.

## Introduction

Combinatorial optimization problems, a subfield of optimization with discrete variables, are ubiquitous in many fields of research. In many cases, we can find a mapping to the decision form of the Ising model with a polynomial number of steps for the NPC (Non-deterministic Polynomial Complete) problem^1–4^. Therefore, many optimization problems can be formulated as Ising models to find the ground state, or the lowest energy configuration. As a result, solving the Ising model has become a general method for solving many NP problems, like partitioning problems^2^, linear programming^1,3,5^, inequality problems^6^, coloring problems^2,7^and so on. However, it is known that the Ising model is an NP-hard (Non-deterministic Polynomial Hard) problem^8^. So, it is difficult but important to find the ground state of the Ising model quickly and accurately.

The Ising model is mainly used in statistical physics and scientific computing. In statistical physics, the Ising model is widely used to study the phase transition phenomenon^9–11^. In scientific computing, the actual combinatorial optimization problem is mapped to the Ising model for finding the ground state in the N spins state space^12–14^. With N spins, there are 2^N^ spin states to search the global minimum energy state, which poses a significant challenge for using conventional computing^15^. Special-purpose hardware devices for the ground state search, known as Ising machines, have recently attracted attention because of their potential to substantially speed up the solution of optimization problems^16^. Various schemes have been proposed and demonstrated for the Ising model, including quantum annealers^17–21^, coherent Ising machine^22–31^, and so on. Limited by current technology, the above methods have difficulties such as large-scale expansion and complicated parameter configuration. Quantum computer may help with these challenges, but related work is still in its infancy^2,32^.

The CMOS implementations^16,33–37^ are easy to integrate and expand, making them a more suitable strategy for mapping and solving large-scale practical Ising model problems. In practice, CMOS Ising machines have advantages such as small size, flexible expansion, high integration, low system power consumption, etc.^36^ Most CMOS chips are based on non-fully connected structures, including lattice graphs^15,33,35,36^, king graphs^34,38–41^, hexagonal graphs^42^, Chimera graphs^43^ and other specific structures^32^. All-to-all connected Ising models have more practical value than sparse ones, but communication and synchronization between the spins can degrade the speed performance in CMOS^16^. As a result, the spin scale of a CMOS chip based on an all-to-all connected topology design is very limited. Non-uniform design limits the widespread adoption of CMOS chips and increases the design cost of ASICs for the Ising model.

AI (Artificial Intelligence) chips have numerous computing resources, which are used for training and inference of various AI algorithms. and serve as valuable resources for solving large-scale problems. Currently, AI chips have solved many problems such as classification, detection, and tracking by virtue of their powerful computing power^44,45^. Commercial AI chips have the characteristics of high energy efficiency, high parallelism, and high scalability. These chips, which are optimized for communication and synchronization, have been used in many large-scale neural network models. The computational architecture of AI chips enables parallel computing, reduced computation time and off-chip storage access through efficient scheduling^46^. Using these computing hardware resources to solve the Ising model with numerous parameters is an extremely effective method.

The paper is organized as follows. In this paper, we propose a new algorithm, Spring-Ising Algorithm, that can solve the all-to-all connected Ising model directly on the AI chip. First, we introduce how Spring-Ising Algorithm inspired by spring vibrations can be used to find the ground state of the Ising model. Then, we design the algorithm as a network structure based on point convolution and residual modules, which implements the solution iteration of the Ising model through point convolution and residual modules. Our method transforms the optimization problem by constructing the Ising model paradigm into the general formula of AI chips calculation and AI chips accelerate Spring-Ising Algorithm for the ground state search. Finally, the network structure is demonstrated on AI chip architecture from Ref.^47^ to solve the Max-cut problem and both numerical and analytical investigation are conducted.

## Modeling

In this chapter, we propose the physical prototype of Spring-Ising Algorithm and how to apply Lagrange's equations to iterate spin states by symplectic method. Spring-Ising Algorithm is inspired by physical phenomena, spring vibrations. The detail of physical prototype is introduced as follows.

1. Spring vibration model.

The Ising model is defined as follows:

1 $$H_{ising} = - \mathop \sum \limits_{1 \le i < j \le N}^{ } J_{ij} \sigma_{i} \sigma_{j} - \mathop \sum \limits_{1 \le i \le N}^{ } h_{i} \sigma_{i}$$

The discrete variable $\sigma_{i}$ is the $i$th Ising spin state such that $\sigma_{i} \in { }\left\{ { - 1,{ } + 1} \right\}$. In Pauli matrices, the variable $\sigma_{i}$ assigns values $\left\{ { - 1,{ } + 1} \right\}$ to spin states $\left\{ { \downarrow ,{ } \uparrow } \right\}$^17^. $J_{ij}$ denotes a coupling coefficient between the $i$th and $j$th spins and $h_{i}$ is an external magnetic coefficient for the $i$th spin. $H_{ising}$ is the total energy of the Ising model and finding the lowest energy of $H_{ising}$ is the target of Ising machines.

**Figure 1:**
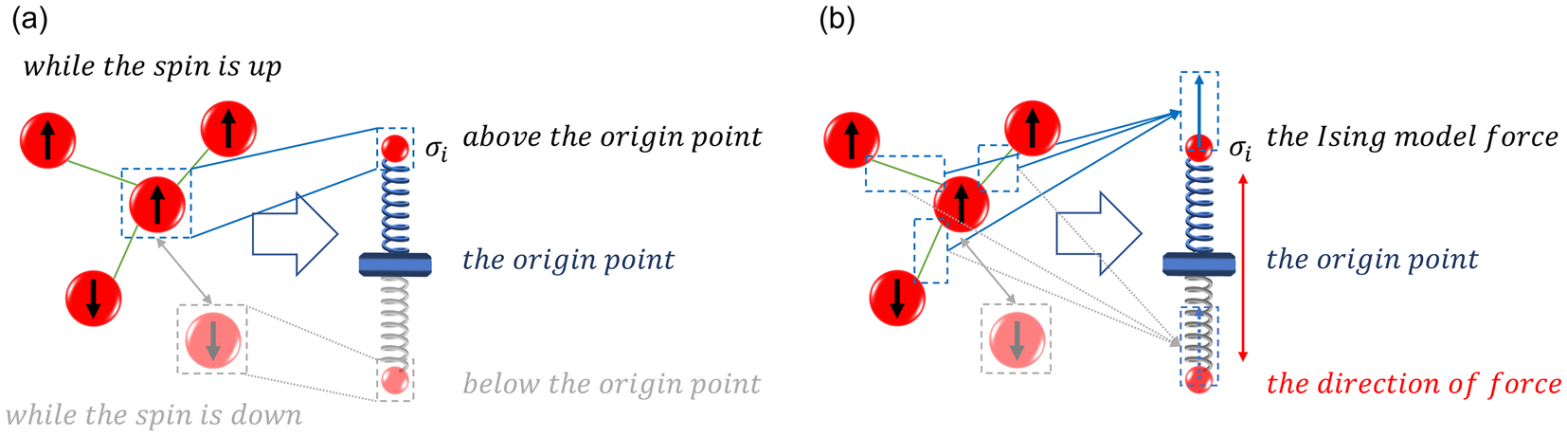
Spring vibration model based on Ising model. The red sphere represents the spin, and the arrow in it indicates the spin state. The four bright red spheres on the upper left represent the four spins mapped by the high-dimensional Ising model. The green connection line between the red spheres represents the coupling relationship. The fuzzy sphere in the gray dashed box represents the opposite spin state of the blue dashed box. The two dashed boxes are used to represent the same spin in two spin states, expressing the two particle positions of the spring model. Correspondingly, the gray part in the spring model is another spin state. (**a**) In the Ising model, the spin state is mapped to the position of the mass point in the spring vibration model. For example, in the blue dashed box, when the spin state is ‘up’, the mass point is positioned above the origin. Conversely, in the blue dashed box, when the spin state is ‘down’, the mass point is positioned under the origin, which is shown in the gray dashed box. (**b**) The distance between the mass point and the origin point is affected by the coupling relationship and the spring.

Inspired by the steady-state analysis of multiple mass-spring system in analytical mechanics, the ground state search method of the Ising model in this paper is designed. Although a spin in the high-dimensional Ising model is affected by multiple spins, there are only two trends in the spin state $\left\{ { - 1,{ } + 1} \right\}$. Therefore, in the modeling, each spin is considered as the mass point moving on a separate one-dimensional system. In Ising model, the state of the $i$th spin $\uparrow \left( \downarrow \right)$ is encoded as a discrete variable corresponding to a value of $+ 1\left( { - 1} \right)$. We regard the discrete variable as the continuous change of the mass point in the macroscopic position, which is defined as the generalized coordinate $q_{i} \in \left[ { - 1,1} \right]$. On this basis, the spring model is designed by considering a mass point connected at an ideal spring with no initial length and the spring force on the mass point is always pointing to one point, called the origin point. As shown in Fig. 1a, the spring is fixed at the origin point, and the other end is the mass point representing the state of spin. Since the initial length of the spring is zero, when the mass point moves away from the origin, it is pulled by the spring. In this model, the mass point is above(below) the origin to represent the spin $\uparrow \left( \downarrow \right)$, and the distance from the origin point to the mass point is represented as a degree of confidence. According to the coupling coefficient and spin state, the Ising model produces a number of forces along a line along the $q_{i}$ axis. Therefore, the direction of the resultant force is also on the $q_{i}$ axis, as shown in Fig. 1b.

**Figure 2:**
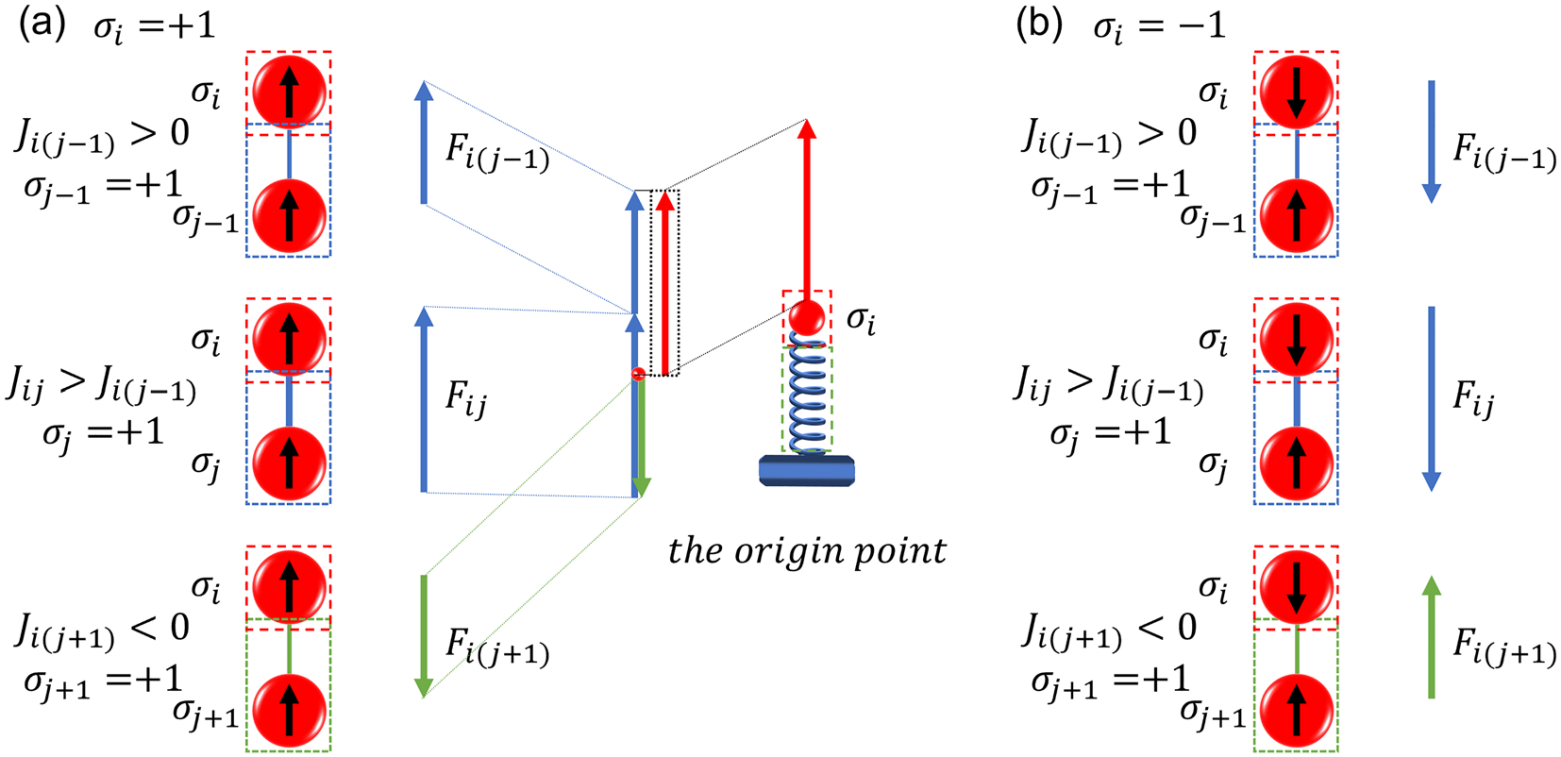
The specific example shows that the coupling relationship between spins affects the external force received on the mass point. $\sigma_{i}$ is the $i$th spin which is regarded as the target spin and $\sigma_{j}$ is the $j$th spin which is regarded as the source spin. A blue line between the spins represents a positive coupling relationship, while a green line represents a negative coupling relationship. The force on the mass point is the resultant force produced by the sum of all coupling relations. (**a**) When the source spin $\sigma_{i}$ is $+ 1$, the coupling relationship produces multiple forces on the mass point $i$. (**b**) When the spin state $\sigma_{i}$ is $- 1$, the direction of the force is opposite.

In the model, while a spin considered as a mass point is called the target spin, the other spins are called the source spins providing external force to the target spins. The magnitude and direction of $F_{i}$ depend on the combined effect of multiple source spins but have nothing to do with the state of the target spin. Figure 2a gives a specific example, when the state of source spin is $+ 1$, if the coupling coefficient is positive, an upward force will be generated. The greater the coupling coefficient, the greater the force generated. In the same way, if the coupling coefficient is negative, a downward force will be generated. When the coupling coefficient is zero, the source spin provides no force. The superposition of all the forces provided by the source spin is the force of the Ising model coupling relationship for the mass point $i$. When the state of origin spin is $- 1$, the direction of the force is opposite, as shown in Fig. 2b.

The generalized coordinate which is introduced by the model is a continuous variable, which means that the magnitude of the force is also affected by the absolute value of the generalized coordinate from the source spin. So, the source spin is represented by the generalized coordinate: $\sigma_{i} \in \left\{ { - 1, + 1} \right\} \to q_{i} \in \left[ { - 1,1} \right]$. When the absolute value of the generalized coordinates is greater, the spring potential energy contained in the spring vibration model is greater. For the Ising model, the greater source spin has a greater overall influence on the system to the target spin and vice versa. Therefore, the discrete Ising model energy in Eq. (1) is set to the continuous Ising model energy in the spring vibration model.

2. Ground state search method.

The spring vibration model can be used to find the ground state of Ising model as follows. This method regards the potential energy of the Ising model as the ordinary potential energy and converts the potential energy of the Ising model into the potential energy of the spring and the kinetic energy of the system. The Ising model energy gradually decreases and transforms into the potential energy of the spring. The Lagrangian equation is constructed as follows:

2 $$L\left( {q_{i} ,\dot{q}_{i} ,t} \right) = \mathop \sum \limits_{i}^{ } m\dot{q}_{i}^{2} - \mathop \sum \limits_{i}^{ } \frac{1}{2}k\left( {q_{i} - q_{0} } \right)^{2} - \zeta H_{Ising} \left( {\varvec{q}} \right)$$

where $m$ is the mass coefficient, $k$ is the elastic coefficient, $q_{0}$ is the generalized coordinate value of the spring origin point and $\zeta$ is the scaling coefficient of the Ising model energy. The three terms of the mass point in Eq. (2) are the kinetic energy term, the spring potential energy term and the continuous Ising model energy term. The continuous Ising model energy term is the energy term of the Ising model derived by replacing the spin states $\sigma_{i}$ with generalized coordinates $q_{i}$. This approach expands the solution space and computational complexity but is more conducive to finding local optimum due to continuous variation. The kinetic energy term acts as an intermediate term in the conversion between the spring potential energy term and the continuous Ising model energy term. In the spring vibration model, the generalized coordinates are independent of $t$. It can be seen from the formula that the movement of the mass points is affected by the potential energy of the spring and the energy of the Ising model. The movement of the mass points is manifested as a continuous vibration on the ideal springs. From another perspective, it can be considered that when the spring is doing simple harmonic motion, a set of external forces are applied from the outside. Affected by the coupling coefficient of the Ising model, the oscillations of the mass points are biased towards the lower Ising model energy.

3. Symplectic method.

Since the size of the Ising model depends on the number of spins, the solution scale is quite large. Therefore, it is very difficult to solve the Lagrangian equation directly and accurately. In this paper, referring to the Hamiltonian and symplectic method^48^, the numerical iterative calculation of the spring vibration model is carried out. The Hamiltonian describes the total energy of the system and can be used to describe the system's dynamic behavior. Symplectic method is a numerical method used to solve Hamilton’s equations and it preserves energy conservation of the system.

According to the definition, the generalized momentum $p_{i}$ is obtained as $\partial L/\partial \dot{q} = m\dot{q}_{i}$. The Hamiltonian of the system is obtained by performing the Legendre transformation on the Lagrangian quantity:

3 $$H\left( {q, p, t} \right) = \mathop \sum \limits_{i} \dot{q}_{i} p_{i} - L\left( {q_{i} ,\dot{q}_{i} ,t} \right) = \mathop \sum \limits_{i} \frac{1}{2}\dot{q}_{i} p_{i} + \mathop \sum \limits_{i} \frac{1}{2}k\left( {q_{i} - q_{0} } \right)^{2} + \zeta H_{Ising} \left( {\varvec{q}} \right)$$

4 $$\begin{gathered} \dot{q}_{i} = \frac{\partial H}{{\partial p_{i} }} \hfill \\ \dot{p}_{i} = - \frac{\partial H}{{\partial q_{i} }} = - k\left( {q_{i} - q_{0} } \right) + \zeta \mathop \sum \limits_{j} J_{ij} q_{j} \hfill \\ \end{gathered}$$

5 $$\begin{gathered} q_{i} \left( {t_{n + 1} } \right) = q_{i} \left( {t_{n} } \right) + {\Delta }\dot{q}_{i} \left( {t_{n} } \right) = q_{i} \left( {t_{n} } \right) + \frac{{\Delta }}{m}p_{i} \left( {t_{n} } \right) \hfill \\ p_{i} \left( {t_{n + 1} } \right) = p_{i} \left( {t_{n} } \right) + {\Delta }\dot{p}_{i} \left( {t_{n} } \right) = p_{i} \left( {t_{n} } \right) - {\Delta }kq_{i} \left( {t_{n} } \right) + \zeta {\Delta }\mathop \sum \limits_{j} J_{ij} q_{j} \left( {t_{n} } \right) \hfill \\ \end{gathered}$$

where $t_{n}$ is the $n$th iteration and ${\Delta }$ is the increment of the time. It can be seen from the above formula that $q_{i} \left( {t_{n} } \right)$ and $p_{i} \left( {t_{n} } \right)$ depend on the value of the previous state. With the iteration of the value, the energy is continuously converted. As the energy of the Ising model decreases, the solution is gradually approaching the ground state of the Ising model. Dimensional issues are not considered in numerical calculations, so parameters can be combined. The Eq. (5) is called the iterative formula of Spring-Ising Algorithm.

The energy contribution of each spin to the overall system in the Ising model energy expression is in a bounded manner because each spin in the Ising model is only in the spin state $\left\{ { - 1,{ } + 1} \right\}$ to contribute to the system energy. In modeling, the generalized coordinate values are with constraints to avoid the appearance that the energy of the whole system is concentrated in few mass points. If there is a sufficient range of energy fluctuations, the system can cross local optimum by local oscillations; but at the same time, if the range of fluctuations is too large, the system cannot stay at any minimum value. So that, the following constraints are added each time $q_{i}$ is updated:

6 $$q_{i} \leftarrow f\left( {q_{i} } \right) = \left\{ {\begin{array}{*{20}l} { - \sqrt 2 ,} \hfill & {q_{i} < - \sqrt 2 } \hfill \\ {q_{i} ,} \hfill & { - \sqrt 2 \le q_{i} \le \sqrt 2 } \hfill \\ {\sqrt 2 ,} \hfill & {q_{i} > \sqrt 2 } \hfill \\ \end{array} } \right.$$

where $f\left( * \right)$ describes the boundary of $q_{i}$. For the spring to vibrate, the boundary is slightly larger than the original setting of Spring vibration model $\left[ { - 1,1} \right]$ so that we set $q_{i} \in \left[ { - \sqrt 2 ,\sqrt 2 } \right]$. Similarly, we simultaneously set $p_{i} \in \left[ { - 2,2} \right]$. After combining the boundary conditions, the equation describes the motion law of the spin.

4. Point convolutional neural network.

**Figure 3:**
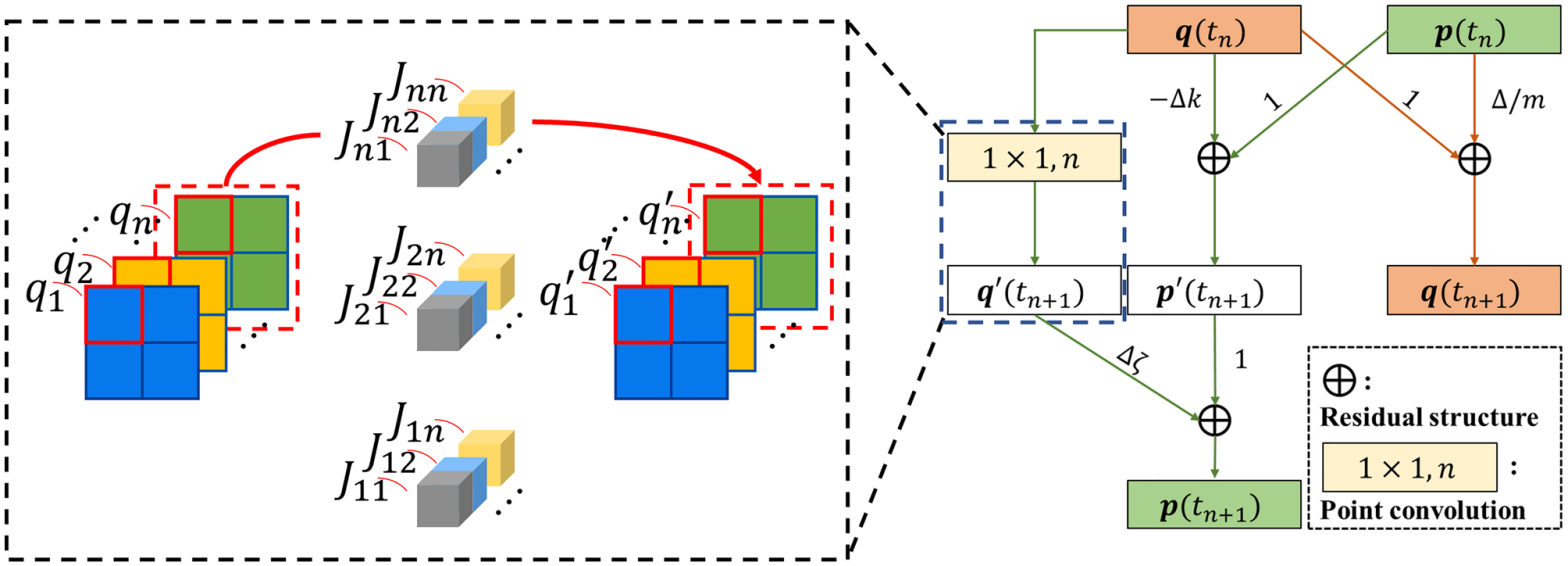
The parallel calculation of the spring vibration model algorithm through the form of point convolution. The size of the feature map affects the number of parallel tests for the algorithm. Using a 2 × 2 feature map is four independent iterative calculations. The value of the feature map is the generalized coordinate value, and the point convolution kernel is the weight data of the Ising model. The $\user2{q^{\prime}}$ and $\user2{p^{\prime}}$ are the temporary variable. On the right is the entire point convolution network architecture.

In the iterative calculation of the algorithm, the computation that consumes the most computational resources is multiplication of $J_{ij}$ and $q_{i} \left( {t_{n} } \right)$. A method of iterative calculation using point convolution to replace the product of vector and matrix is proposed, so that the algorithm can be used in high-bandwidth computing chips, like GPU and AI chip. Point convolution is a point-by-point 2D convolution operation on an image by the $1 \times 1$ convolution kernel. A pixel point in an image is composed of component or feature information, which means that each pixel point can be represented by a vector, this is known as channel information. This type of image is also called the feature map. Point convolution is widely used in lightweight neural networks, and many hardware architectures have been designed to accelerate computation by designing schemes that optimize the computational mapping of point convolution^45–47,49^. Figure 3 shows the way of turning the iterative equation into the neural network architecture computation. If the Ising model has $n$ spins, a single point convolution kernel has $n$ channels, corresponding to the coupling coefficients (including self-coupling) of a single spin to the other spins. Each of the $n$ spins correspond to $n$ point convolution kernels, forming the $1 \times 1 \times n \times n$ weights (convolutional kernels size $1 \times 1$, number of channels $n,$ number of convolutional kernels $n$) corresponding to the Ising model coupling coefficients $J$. $q_{i} \left( {t_{n} } \right)$ of a single test is assigned at fixed coordinate of the feature map, meaning that the size of the feature map is equal to the number of simultaneous test cases. Choosing a $1 \times 1$ feature map for a single Spring-Ising Algorithm test, and a feature map size of $2 \times 2$ as illustrated in Fig. 3, four mutually independent tests of the Spring-Ising Algorithm are performed simultaneously. The rest of the architecture is the addition, which can be completed through the residual structure in the neural network and is supported in mainstream AI chips. The method uses $n$ convolutional kernels that can be computed in parallel at the same time, which reflects the parallelism of the chip's computation.

## Result

In this chapter, we show the experimental results based on the spring vibration model. Next, we introduce how to implement the above algorithm through point convolution and residual network and implement it on the CASSANN-v2 architecture.

To demonstrate the effect of Eq. (1), the algorithm is tested on the K2000 benchmark instance, which is a random undirected graph with 2000 vertices and 1,999,000 edges^23^. The K2000 benchmark instance has been widely employed for evaluating the performance of the Ising model in solving maximum-cut problems (MAX-CUT) in previous studies^23,50,51^.

1. Qualitative results.

**Figure 4:**
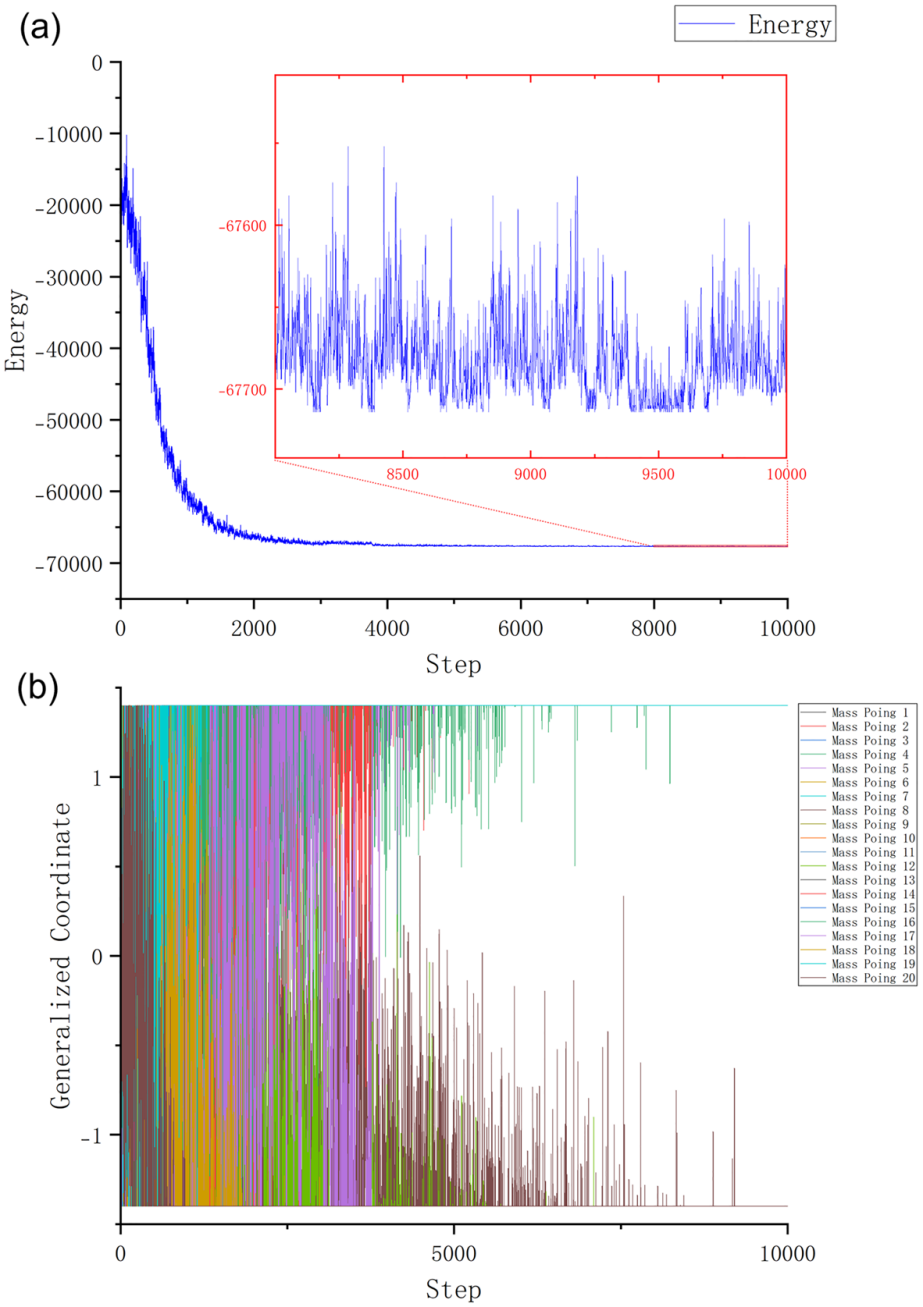
The spring vibration model algorithm on the K2000 in 10,000 iterations. The parameter configuration is as follows: k = 0.5, ζ = 0.8ζ_0_ →10ζ_0_, Δ = 0.2, m = 1. (**a**) The energy change curve of the Ising model. The mass point positions in Spring-Ising Algorithm are initialized near the origin, so the energy starts from 0 and decreases rapidly. Before Step = 2000, the energy is descending in a violent shock. After that, vibrate slightly to search for the energy minimum. (**b**) Vibration of the mass points (the first twenty). The densely populated regions of the graph result from the oscillations of multiple mass points. As the system completes the initial search, it tends to be stable. While most of the mass points become stable, only a few of them continue to perform local searches (e.g., after Step = 5000).

The mass point vibration result of running the spring vibration model algorithm in 10,000 iterations is shown in Fig. 4. The 2000 vertices of K2000 correspond to the 2000 generalized coordinates of the Spring-Ising Algorithm, and for visualization purposes, the first twenty vertices in K2000 are selected in Fig. 4. During the early stage of the algorithm, as the mass points are initialized at origin and given only a small disturbance, the energy of the Ising model experiences a gradual decline. It can be clearly seen in the figure that, the polylines are very dense, which means that the mass points are oscillating violently. In this time, the energy of Ising model is also rapidly oscillating and declining. In the middle, many mass points gradually move towards the boundary, having reached lower energy points. Finally, only a few mass points continue to oscillate in search of the optimal result. The energy of the Ising model has approached the ground state and the details of the energy changes are shown in the inset of Fig. 4(a). It is evident that the flips of a few spin states lead to fluctuations in the Ising energy.

2. Quantitative results.

It can be easily predicted that the potential energy of the spring is lost within the limitation of the boundary conditions as time progresses. Therefore, in the later stages of evolution, it is necessary to compensate for the lost energy. To further search the ground state of Ising model accurately, Spring-Ising Algorithm introduces the concept of energy dynamic balance to increase the energy proportion of Ising model and improve the search efficiency. To compensate for the energy loss, Spring-Ising Algorithm sets the $\zeta$ as a linear variable $\zeta \left( {t_{n} } \right)$. To reduce the complexity of the algorithm, this variable is regarded as a constant in the calculation of the Lagrangian equation, which means that the time-varying effect in the Lagrange equation is not considered. Through further analysis and solution of this equation, the ground state finding of the Ising model system is obtained.

**Figure 5:**
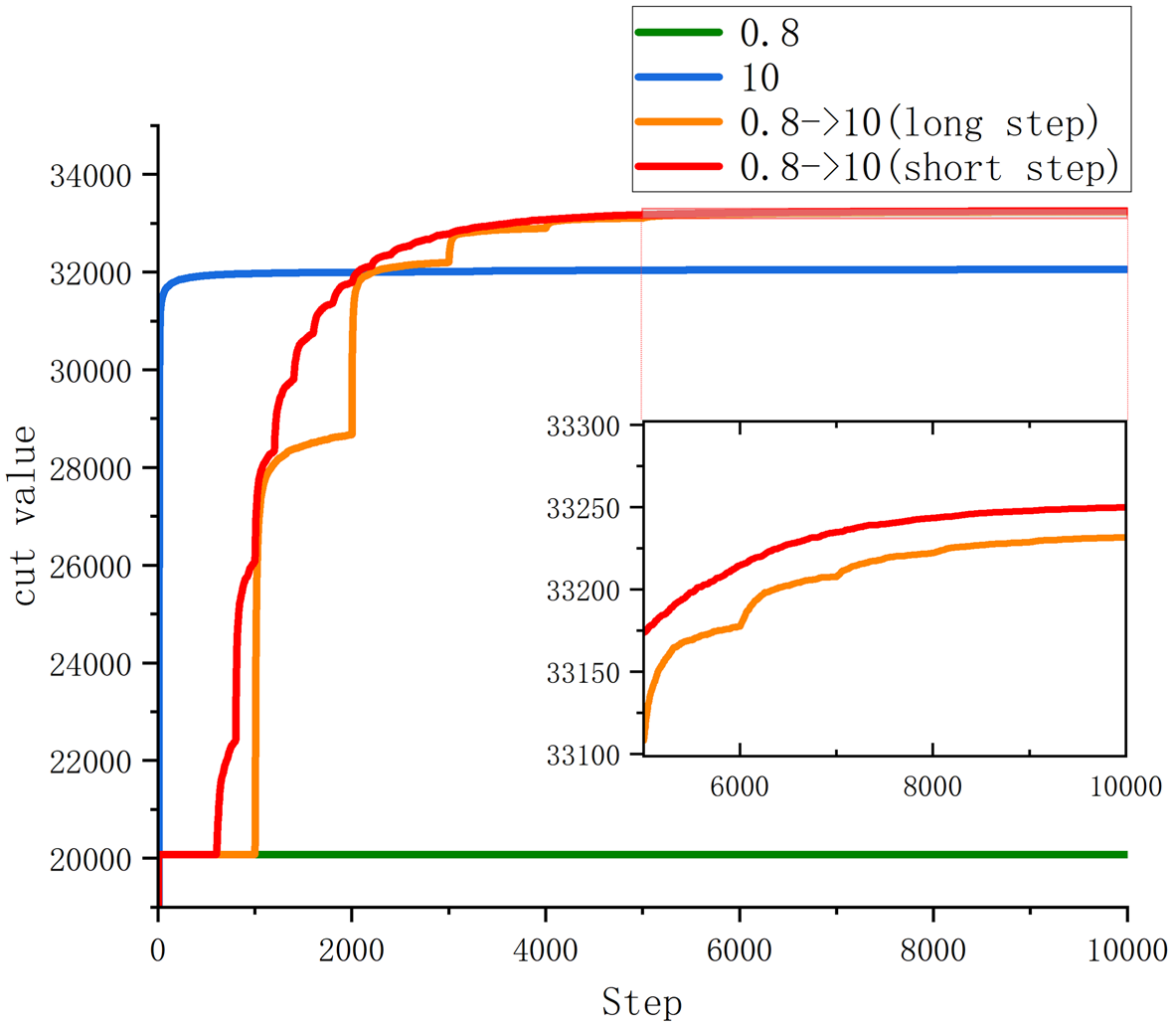
The effect of different ζ on the average results of K2000. ζ_0_ is the base value. ζ_0_ = 0.05. The first and second sets of data (green curve and blue curve) indicate that the current ζ is fixed at 0.8ζ_0_ or 10ζ_0_, respectively. The third (Step = 1000) and fourth (Step = 200) set of data (orange curve and red curve) indicates that the ζ is set from 0.8ζ_0_ to 10ζ_0_ with different step lengths.

This test is based on the same small disturbance for initializing with different strategies of $\zeta$. As shown in Fig. 5, no matter what the value of ζ is fixed, the ground state search of the Ising model is easy to fall into a local optimum. Although the larger $\zeta$ quickly leads to better local optimum (the blue line), it is difficult to search further to get better results. By gradually changing the value of ζ, further searches can be performed after the spring model has entered local stability. The red line and the orange line can be clearly seen each time steady state is established and further searches. This result is very similar to sufficiently slow cooling in simulated annealing. When the step length is short enough, better search results can be obtained. We tested the amount of runtime increase introduced by the computational volume of the energy dynamic balance. The results of 20 rounds of tests with 2000 spins of Ising model, 1000 test cases, and 10,000 iterations are as follows, the average time taken without the introduction of energy dynamic balance is 11.95991 s and the average time taken with the introduction of energy dynamic balance is 12.10152 s. Therefore, the introduction of energy dynamic balance in this test increased the computational time consumption by 1.17% on average. To assess the performance of energy dynamic balance, we tested different coupling relationships of Ising model in 1000 independent experiments, as shown in Table 1. We consider Möbius ladder graph, five different connection graphs from the Gset dataset and K2000, all of them with 2000 spins. The statistics are split into two increments based on the coupling sparsity, because the energy term of the Ising model in Eq. (3) is not the same as the ratio of the whole energy system at different sparsities. So, the increment is set smaller for the K2000 instances where the K2000 Ising model energy is more influential. Due to the antiferromagnetic coupling to neighboring spins, Ising model of Möbius ladder graph is difficult to find the ground state^28^. Gset^52^ consists of toroidal, planar, and random graphs with weights taking the values 1, or − 1, where G22 and G27 are random graphs, G32 is a toroidal graph, and G35 and G39 are planar graphs. K2000 is the fully connected random graph mentioned above. The results in Table 1 show that the method can be extended and applied to the Ising model under various structures.

**Table 1:** Results of Spring-Ising Algorithm for Ising model with different coupling relationships based on the energy dynamic balance approach in 1000 independent experiments.

	Möebius ladder	Gset-G22	Gset-G27	Gset-G32	Gset-G35	Gset-G39	K2000
Spins	2000	2000	2000	2000	2000	2000	2000
Connectivity	3000	19,990	19,990	4000	11,778	11,778	1,999,000
Weight	− 1	− 1	+ 1, − 1	+ 1, − 1	− 1	+ 1, − 1	+ 1, − 1
sparsity	0.0015	0.0100	0.0100	0.0020	0.0059	0.0059	1.0000
Increment	0.01/200 steps	0.01/200 steps	0.01/200 steps	0.01/200 steps	0.01/200 steps	0.01/200 steps	0.001/200 steps
Best known	− 2000	− 6726	− 6724	− 2778	− 3552	− 4771	− 67,714
Best result	− 2000	− 6726	− 6724	− 2774	− 3540	− 4748	− 67,714
Mean result	− 1979.2	− 6689.26	− 6690.01	− 2741.64	− 3499.48	− 4705.68	− 67,497.52

Best known refers to the lowest energy yet reported with a conventional algorithm. Best result and Mean result refers to the statistical Ising energy results by Spring-Ising Algorithm.

**Figure 6:**
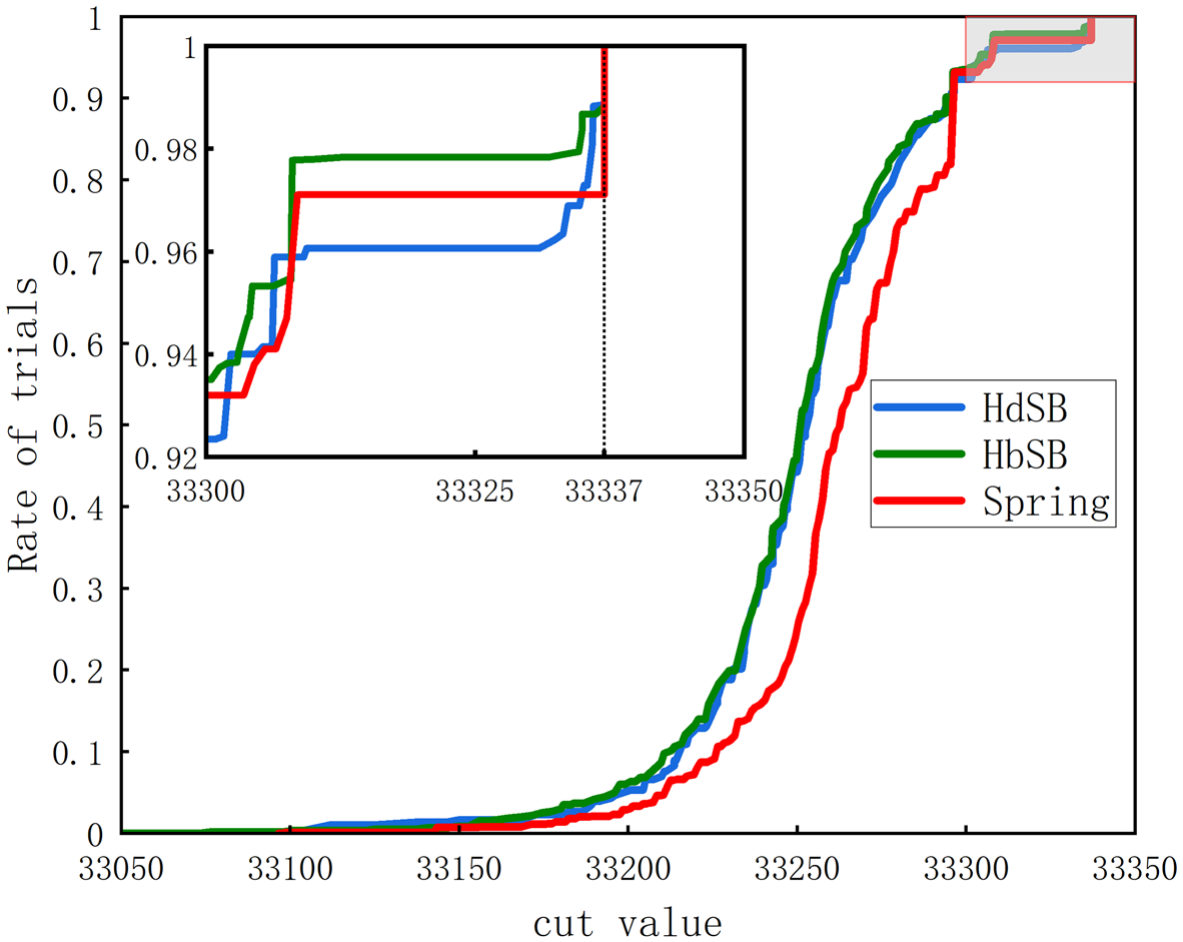
The spring vibration model algorithm cumulative distribution of cut values C of the K2000 compared to HdSB and HbSB. The red curve is the result of the Spring-Ising Algorithm. The inset is the magnification around the best-known cut value. The red curve illustrates that the Spring-Ising Algorithm has better suboptimal distribution results and more optimal values than HdSB and HbSB for the overall search results.

The probability density function is an important way to judge the performance of algorithms for solving Ising models. Figure 6 shows the cumulative distributions of the cut value of the K2000. The results obtained by the proposed algorithm are compared with those obtained by the HdSB and HbSB algorithms which are partially similar under different modeling approaches^50^. It shows that the spring vibration model algorithm can search for better cut value within the specified number of steps. The inset shows that the algorithm can more effectively find the optimal value. The number of optimal solutions accounts for 2.9% of all solutions. In contrast, HbSB and HdSB only achieve about 1.2%.

3. Hardware implementation.

The test platform of this algorithm is a personal computer (Intel 8700K and NVIDIA GeForce RTX 2080 Ti) and the AI architecture (CNN accelerator) developed by Institute of Semiconductors, CAS, named CASSANN-v2^47^. Using GeForce RTX 2080 Ti in the PyTorch framework, with 2000 spins and 1000 independent tests, the calculation time is 9.95 s for 10,000 steps, which means that the sample time of 10,000-step tests is 9.95 ms. But when there are 100 independent tests, the sample time is 2.30 ms for 10,000 steps. The GPU exhibits shorter average single-sample test time with more independent tests. By the AI architecture, when 2000 spins and 49 independent tests (7 $\times$ 7 feature map) are performed, the calculation time is 381.15 ms for 10,000 steps, which means that the sample time of 10,000-step tests is 7.78 ms.

## Discussion

We have proposed and implemented an Algorithm, which is suitable for hardware computing to find the ground state of the Ising model. In Eq. (6), the introduction of the boundary of $q_{i}$ is something that can significantly impact on the energy of the system. Setting the boundary is necessary because unbounded $q_{i}$ would cause the continuous Ising model energy term to decrease indefinitely, leading to an infinite increase in the spring term. This is simply confirmed by calculations and experiments. When the boundary is set, each constraint update to the generalized coordinates $q_{i}$ is a nonlinear operation. The non-linear operation is essential for encoding the quantized Ising spins using continuous variables, similar to techniques like the phase-sensitive amplifier^23^, the Kerr-nonlinear parametric oscillators in simulated bifurcation^48^. The non-linear operation involving restricted boundaries ensures an accurate representation of the Ising model energy by the continuous Ising model energy term. This nonlinear operation described in the paper is both straightforward and efficient. However, there are better nonlinear methods to achieve the corresponding effect among neural networks. Future work will involve testing these methods and integrating them into the Spring-Ising Algorithm. Since the activation function is one of the fundamental components of an AI chip.

However, during the experiments, there is still a problem that the result keeps converging to a local optimum. In the algorithm, the oscillatory search for the Ising model ground state is the original design intention to further obtain more optimal solutions. Simulated bifurcation introduces the thermal fluctuation^50^ to escape from local optimum, which is an effective method. Similarly, in this paper, an external method is introduced to improve the search efficiency, referred to as the concept of energy dynamic balance. The method increases the scaling coefficient of the Ising model energy ζ, thus compensating for the energy loss due to the boundary conditions. The most important benefit of introducing this method is that it doesn't add too much computational effort for the hardware computing. By using weight coupling, it is still feasible to compute in the form of pointwise convolution by AI chips. This also implies that it is indeed possible to preprocess Ising models with different graph structures making it possible to search for energy minima more efficiently using this algorithm. This will be one of the directions for future work.

## Methods

In this paper, we introduce a novel spring-vibration model and propose the Spring-Ising algorithm, designed for the efficient ground state search of Ising models through the utilization of a point convolutional neural network. The Spring-Ising algorithm can be mapped to a GPU or AI chip to accelerate the ground state search of the Ising model by the fundamental structural framework of the neural network. The Spring-Ising algorithm has better suboptimal distribution results and more optimal values than HdSB and HbSB for the overall search results when tested on the K2000 dataset.

1. Numerical iteration.

The Spring-Ising Algorithm is to regard the spin of the Ising model as $q$ and the coupling coefficient weight as $J$. The ground state search process of the Ising model is conducted in conjunction with the oscillation of mass points. Utilizing the spring vibration model as a foundation, we construct an equation that combines vibrations with the Ising model. In Algorithm 1, the pseudo-code illustrates the iterative computational procedure of the algorithm from initialization to sampling.

**Algorithm 1:**
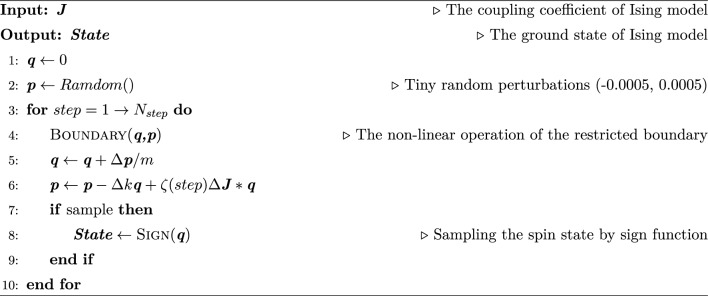
The iterative computational procedure of the Spring-Ising Algorithm.

The initial step involves setting $q$ to $0$ and $p$ to a value in the vicinity of $0$. The $p$ values are generated as random numbers ranging from − 0.0005 to 0.0005, and they do not undergo any manual processing. $N_{step}$ represents the desired number of iteration rounds, which is related to the number of iterations in the Ising model. Increasing the number of iterations during testing results in obtaining more optimal values and improved averages. The ‘Boundary' function performs the nonlinear operation that constrains the range of generalized coordinates, ensuring that $q_{i} \in \left[ { - \sqrt 2 ,\sqrt 2 } \right],{ } p_{i} \in \left[ { - 2,2} \right]$. ${\Delta }$, $k$ and $\zeta$ are independent adjustable variables. $\zeta \left( {t_{step} } \right)$ is a function that is linearly related to the number of iterations. For simplicity in calculation, $\zeta \left( {t_{step} } \right)$ is set as a piecewise constant function. The final step involves sampling $q$ to obtain the spin states of the Ising model. The ‘Sign’ function is used to obtain the sign value of $q$, corresponding to the spin state $\left\{ { - 1,{ } + 1} \right\}$.

2. Hardware implementation.

In the case of an Ising model with $n$ spins, the generalized coordinates $q$ are mapped to feature maps. The number of pixels in feature map corresponds to the number of simultaneous iterations. The coupling coefficient matrix of the Ising model is mapped to the point convolution kernel. Divide the $J$ into $n$ 1 × 1 convolution kernels with $n$ channels by row. Through the residual structure, the addition operation required in the algorithm is completed. By continuously calling this network structure (Fig. 3), the numerical calculation of $q$ and $p$ in the Eq. (8) is updated. After an artificially set time step or calculation time, the $q$ is sampled, which is the current low energy state of the Ising model. In the CASSANN-v2 deployment, an 8-bit quantization scheme was used, including $q$, $p$, $J$.

## Data Availability

The data that support the findings of this study are available from the corresponding author upon reasonable request.

## Code availability

The code used in this work is available from the corresponding author upon reasonable request.
